# Endoscopic Diagnosis of Biliary Stricture Combined with Digital Cholangioscope: A Case Series

**DOI:** 10.3390/healthcare10010012

**Published:** 2021-12-22

**Authors:** Seiichiro Fukuhara, Eisuke Iwasaki, Atsuto Kayashima, Yujiro Machida, Hiroki Tamagawa, Shintaro Kawasaki, Masayasu Horibe, Shutaro Hori, Yuta Abe, Minoru Kitago, Haruhiko Ogata, Takanori Kanai

**Affiliations:** 1 Center for Diagnostic and Therapeutic Endoscopy, Keio University School of Medicine, Tokyo 160-8582, Japan; fukuharas@luck.ocn.ne.jp (S.F.); hogata@z8.keio.jp (H.O.); 2 National Hospital Organization Tokyo Medical Center, Division of Gastroenterology and Hepatology, Tokyo 152-8902, Japan; arbor.caeli@gmail.com; 3 Division of Gastroenterology and Hepatology, Department of Internal Medicine, Keio University School of Medicine, Tokyo 160-8582, Japan; shimauma@LIVE.COM (A.K.); machiyumachiyu@gmail.com (Y.M.); thememorialworld@hotmail.com (S.K.); masayasu.horibe@gmail.com (M.H.); takagast@z2.keio.jp (T.K.); 4 Department of Surgery, Keio University School of Medicine, Tokyo 160-8582, Japan; shutaro.hori@gmail.com (S.H.); abey3666@gmail.com (Y.A.); dragonpegasus427@gmail.com (M.K.)

**Keywords:** biliary stenosis, digital cholangioscopy, biopsy, endoscopic retrograde cholangiopancreatography

## Abstract

The endoscopic diagnosis of biliary tract lesions is applied as a non-invasive method; however, its diagnostic accuracy is not yet high. Moreover, digital cholangioscopy is used for directly visualizing the inside of the bile duct, resulting in a more precise biopsy. We present the case series of the outcomes of diagnosis using digital cholangioscopy in patients who underwent cholangioscopy for the evaluation of biliary stenosis in our department between January 2014 and March 2021. The controls were those who underwent a biopsy for biliary stenosis with conventional endoscopic retrograde cholangiopancreatography (ERCP). Background data for each case were collected, and the clinical outcomes by biopsy were evaluated, focusing on the accuracy of the diagnosis. Cholangioscopy was performed in 15 cases, while a conventional biopsy by ERCP was performed in 172 cases. Nine of 15 cases (60.0%) were diagnosed with cholangiocarcinoma. The number of specimens obtained through conventional ERCP and cholangioscopy was 2.5 ± 1.3 and 3.3 ± 1.5, respectively (*p* = 0.043). The diagnostic accuracy of conventional ERCP and cholangioscopy were 65.7% (113 of 172 cases) and 100%, respectively, which was significantly higher in the group with cholangioscopy. Digital cholangioscopy is useful when the diagnosis of the biliary stricture using the conventional ERCP method is difficult.

## 1. Introduction

Advances in endoscopic technology have improved the diagnostic techniques for biliary tract diseases. However, because of the narrow lumen and the approach through the papilla of Vater, the diagnosis of biliary tract diseases is often more difficult than that of gastrointestinal tract diseases, where direct observation and biopsy under direct vision are possible. Thus, the diagnoses of biliary tract diseases rely on cholangiography and fluoroscopic biopsy performed via endoscopic retrograde cholangiopancreatography (ERCP). However, this method is limited by its low sensitivity. A systematic review on the detection of malignant biliary stricture using ERCP revealed that the pooled sensitivity of brushing cytology and forceps biopsy was 45% and 48.1%, respectively [1]. The combination of these two methods resulted in a sensitivity of only 59.4%. In addition, it is difficult to make definitive diagnosis in about 20% of cases relating to the biliary stricture [2]. In such cases, surgery is sometimes required for a definitive diagnosis. Thus, a more accurate diagnosis of biliary tract diseases is important to avoid unnecessary surgical procedures and optimize the patient’s quality of life.

Oral cholangioscopy is a useful endoscopic approach for the diagnosis of biliary tract diseases because it allows the direct observation and biopsy of the bile ducts. Recently, the development of electronic and digital cholangioscopes has allowed clinicians to perform biopsies while viewing endoscopic images directly in the biliary tract. For indeterminate biliary strictures, the diagnostic sensitivity of conventional ERCP biopsy under fluoroscopy was 40–87%, while that of cholangioscopy was 65–100% [3,4,5,6,7,8,9,10]. Cholangioscopy has been considered to be challenging because of its difficulty to use, and its high invasiveness, operability, and durability. However, recent improvements have made it possible to obtain high-resolution images, improve the angle of operation, and perform operations such as aspiration, water supply, and biopsy simultaneously. Significantly, the SpyGlass system (Boston Scientific, Natick, MA, USA) is a single-operator POCPS technique with two independent dedicated irrigation channels and four-way tip deflection for complete observation. These innovations are expected to greatly improve the diagnosis of biliary tract diseases.

Therefore, the present study aimed to examine the outcomes of diagnoses performed with cholangioscopy by presenting a case series that we have experienced in our hospital.

## 2. Materials and Methods

### 2.1. Study Design

This retrospective case series included patients with biliary stenosis who underwent a biopsy via cholangioscopy at our university hospital from January 2014 to March 2021. Patients with biliary stenosis who underwent conventional biopsy with ERCP during the same period were identified as controls. Cases in which the cause of bile duct stenosis was thought to be common bile duct stones or compression by lymph nodes or other lesions outside the bile duct were excluded. This study was performed in accordance with the 2008 revision of the Declaration of Helsinki. The study protocol was approved by our institutional review board (approved on 25 September 2015, no. 20150245).

### 2.2. ERCP Procedure

The ERCP procedure was mainly performed using a side-viewing endoscope (TJF-260V, JF-260V, or TJF-290V; Olympus Medical Systems, Tokyo, Japan). A side-viewing endoscope was inserted carefully. A catheter (ERCP-Katheter, 0120211; MTW Endoskopie, Wesel, Germany) was then advanced into the common bile duct with a 0.025-inch hydrophilic guidewire (VisiGlide 2™; Olympus Medical Systems, Tokyo, Japan). If each deep cannulation was difficult, another 0.025-inch hydrophilic guidewire (NaviPro™; Boston Scientific Japan, Tokyo, Japan) was used. After the injection of contrast medium into the common bile duct, endobiliary forceps biopsy was performed for biliary stenosis using direct biopsy forceps (Radial Jaw™; Olympus Medical Systems, Tokyo, Japan) under fluoroscopy. Endoscopic sphincterotomy (EST) was performed in some cases as a small to medium-sized incision.

### 2.3. Cholangioscope Procedure

In cases with cholangioscopy, EST was performed before insertion with a sphincterotome catheter (CleverCut3V; Olympus Medical System, Tokyo, Japan). After EST was performed as a medium-sized incision, a cholangioscope (SpyGlass DS™; Boston Scientific Japan, Tokyo, Japan, or CHF-B260; Olympus Medical System, Tokyo, Japan) was inserted into the common bile duct along with the guidewire. Biliary stenosis was recognized by the injection of normal saline. When a SpyGlass DS was used, the biopsy was performed using forceps (Spybite™; Boston Scientific Japan, Tokyo, Japan) with direct endoscopic visualization. In cases with CHF-B260, tissue specimen was obtained by using forceps (FB-45Q; Olympus Medical System, Tokyo, Japan). The number of the tissue samples depended on the operator.

### 2.4. Study Outcomes

The primary outcome measure was the diagnostic accuracy of the endobiliary biopsy. Secondary outcomes included the patients’ characteristics (age, sex, tumor size, and location) and endoscopic procedure details, such as the number of specimens obtained by the biopsies. The accuracy of the diagnosis was defined by the histopathology of the surgical specimen or the clinical course at least six months after the endoscopic procedure by repeated imaging and blood tests. In addition, endoscopic diagnosis was defined as a combined diagnosis of the cholangiography and histological evaluation obtained by using an endoscopic biopsy.

### 2.5. Statistical Analysis

All continuous values are presented as mean ± standard deviation. The unpaired Student’s *t*-test or one-way analysis of variance was used to evaluate the statistical significance of differences. Fisher’s exact test was used to evaluate categorical data. Statistical significance was considered for two-sided *p*-values of <0.05. All statistical analyses were performed using SPSS software version 26.0 for Windows (SPSS Japan, Tokyo, Japan).

## 3. Results

### 3.1. Patients’ Clinical Features

Table 1 shows the patients’ characteristics. During this period, the examination for conventional biopsy using ERCP was performed in 172 cases, while it was performed in 15 cases with cholangioscopy. The median ages in the group with conventional biopsy using ERCP and cholangioscopy were 69.7 ± 10.4 and 72.2 ± 11.2 years, respectively. In both groups, males were more dominant than females. By category, the proportion of males was 62.6% (87/139) for malignant lesions and 70.8% (34/48) for benign lesions, respectively (*p* = 0.30). Furthermore, it was 65.1% (69/106) for lesions located at the distal region and 64.2% (52/81) for lesions located at the hilar region, respectively (*p* = 0.90). The location of the targeted biliary stenosis was slightly more dominant in the distal region than in the hilar region. The proportion of malignancy in the group with conventional biopsy by ERCP and cholangioscopy was 75.6% (130 of 172 cases) and 60.0% (nine of 15 cases), respectively. There were no significant differences in each category.

### 3.2. Details of Patients with Cholangioscopy for Biliary Stenosis

**Figure 1:**
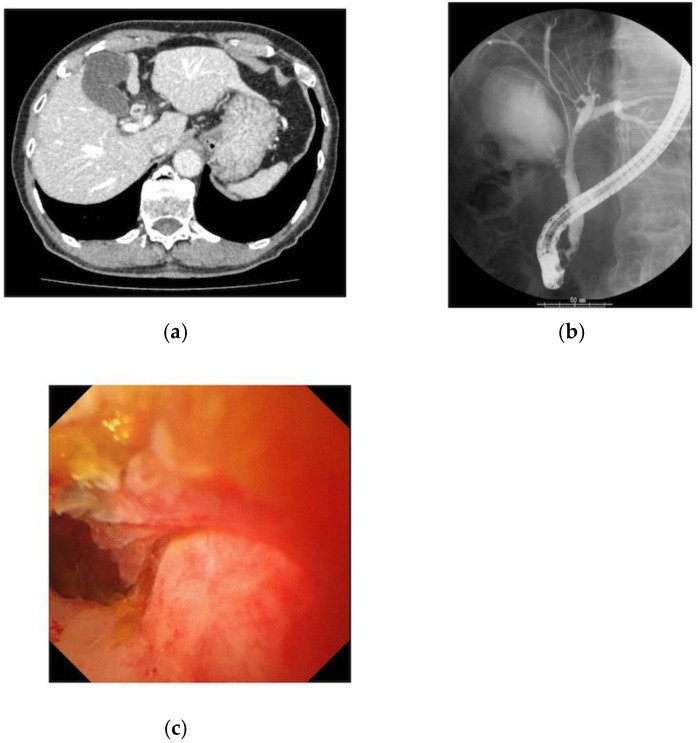
Images of the case with bile wall thickness which was finally diagnosed as IgG4SC: (**a**) The CT scan revealed wall thickening in the common bile duct, with nodular thickening of the bile ducts in the hilar region; (**b**) the ERCP showed that the wall of the bile duct was hard, and the mucosal edge was rough from the cystic duct to the hilar region of the bile duct; (**c**) cholangioscopy showed rough mucosa with irregular papillary elevation.

The details of each patient who underwent a cholangioscopy are outlined in Table 2. A SpyGlassDS was applied as a cholangioscope in 11 of 15 cases (73.3%). Out of 15 patients, nine (60.0%) were patients with cholangiocarcinoma, whereas six had benign stenoses, such as immunoglobulin G4 sclerosing cholangitis (IgG4SC) and primary sclerosing cholangitis (PSC). In 11 out of 15 cases (73.3%), the cholangioscopy was performed for further definitive diagnosis, since the condition was difficult to diagnose using conventional ERCP. In the remaining four cases, a cholangioscopy was performed to evaluate the extension of the biliary lesion in addition to the diagnosis. The diagnostic accuracy of biopsy specimens was 100%. In all four cases performed for the purpose of tumor extension range, the diagnosis was made accurately. In one of the cases, it was difficult to distinguish IgG4SC from cholangiocarcinoma given that it was pathologically benign, though it was finally diagnosed as IgG4SC after surgery (Figure 1).

### 3.3. Outcomes of Diagnosis using Conventional ERCP and Cholangioscopy

**Figure 2:**
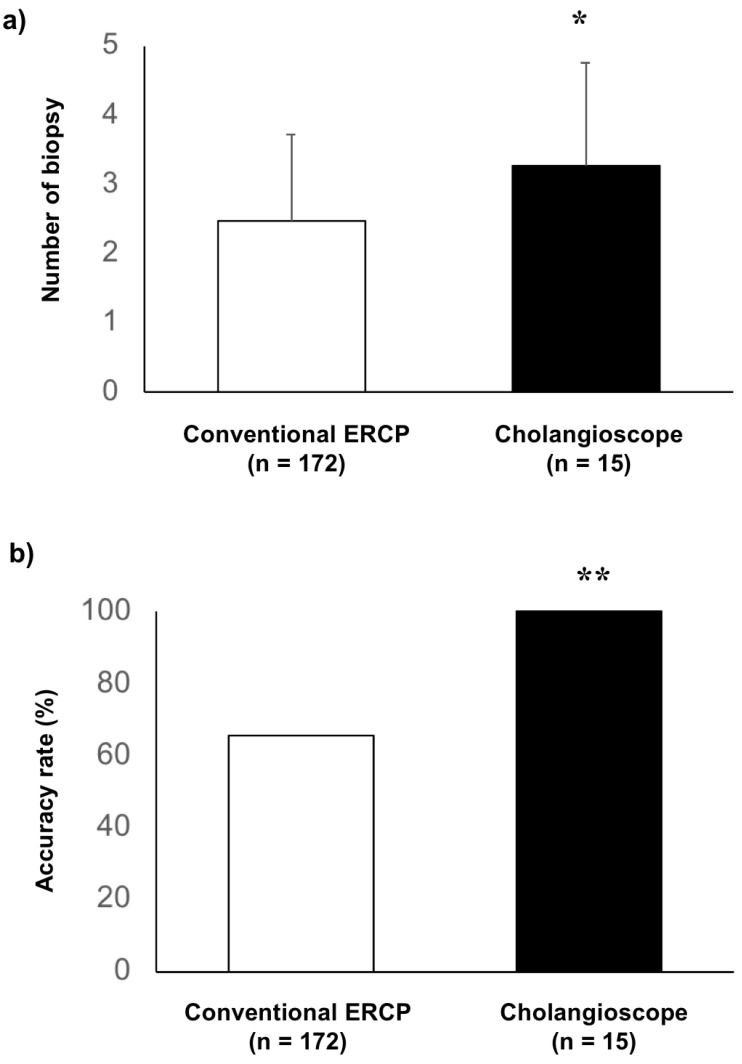
Comparison of biopsy outcomes in cases with conventional ERCP and cholangioscopy: (**a**) the number of biopsy specimens in cases with cholangioscopy (3.3 ± 1.5) was significantly higher than that with conventional ERCP on average, with a significant change of 2.5 ± 1.3 in cases with cholangioscopy (*p* = 0.043); * indicates *p* < 0.05; (**b**) the accuracy rate in cases with cholangioscopy was significantly higher than that in cases with conventional ERCP (100% and 65.7%, *p* = 0.003); ** indicates *p* < 0.01.

The outcomes of the biopsy for biliary stenosis are shown in Figure 2. The number of specimens obtained using conventional ERCP and cholangioscopy was 2.5 ± 1.3 and 3.3 ± 1.5, respectively (Figure 2a, *p* = 0.043). The diagnostic accuracies of conventional ERCP and cholangioscopy were 65.7% (113 of 172 cases) and 100%, respectively, which was significantly higher in the group with cholangioscopy (Figure 2b, *p* = 0.003).

## 4. Discussion

In the present retrospective study, we analyzed 15 patients who underwent cholangioscopy for the evaluation of biliary stenosis as a case series. Compared with the 172 cases that underwent conventional biopsy using ERCP for the diagnosis of biliary stenosis, this study revealed that the diagnostic accuracy was superior in cases with cholangioscopy. Since most cases with cholangioscopy were difficult to diagnose via conventional endoscopic procedures, it was suggested that biliary biopsy associated with cholangioscopy was useful for the diagnosis in the present case series.

Differentiation between benign and malignant lesions presenting with bile duct stenosis and tumor localization is important to avoid unnecessary surgery and to determine the extent of surgery. However, it is often difficult to make an accurate diagnosis using imaging. The diagnostic usefulness of oral cholangioscopy for the diagnosis of bile duct stricture lesions has recently been evaluated in many studies. In particular, with the switch from using a fiberscope to a videoscope, the image accuracy has improved dramatically. The diagnostic sensitivity for malignant lesions is 78–83% for endoscopic findings and 49–86% for biopsy under direct visualization, which is higher than that of conventional ERCP cytology and biopsy [7,11,12,13,14,15,16]. Moreover, cholangioscopy can provide a positive diagnosis in 77–85% of cases in which conventional ERCP failed to provide a diagnosis [12,14]. In addition, it is important to diagnose the horizontal extent of cholangiocarcinoma to determine the extent of the resection, since it has been reported that adding the findings of oral cholangioscopy to those of cholangiography improves the accuracy [17]. In the present study, our data showed that the diagnostic accuracy was significantly better in the cases where cholangioscopy was used than in those using conventional biopsy with ERCP. In detail, it was effective at diagnosing biliary tract lesions and identifying the extent of the lesions. Though there was a higher proportion of male patients in this study, there was no significant difference by lesion or location. This proportion may reflect the epidemiological features of male prevalence with IgG4SC and cholangiocarcinoma. Meanwhile, the number of biopsies was significantly higher in the cases with cholangioscopy than in those with conventional ERCP. However, it cannot be ruled out whether the number of biopsy specimens contributes to the positive diagnosis rate in cases with cholangioscopy. This may be due to the fact that most cases with cholangioscopy could not be diagnosed in the initial session performed using the conventional method, and the number of biopsies was increased for a more careful and reliable diagnosis. However, the difference in the number was not so large, and the biopsy forceps for cases using cholangioscopy were smaller than those used in the conventional method. Therefore, the use of cholangioscopy allows for a more accurate biopsy through the confirmation of the biliary lesion. Furthermore, the combination of conventional ERCP with oral cholangioscopy improves the diagnostic accuracy for biliary stricture, as shown in the present case series.

Diagnosis via cholangioscopy has previously relied on identifying the pathology using a biopsy in many cases. Benign bile duct lesions often have a wide variety of markers, which vary depending on the activity of the disease, making it difficult to obtain specific markers for each disease. Moreover, benign biliary tract lesions include a low, uniform granular mucosa with low length, uneven surface findings, and scarring. Meanwhile, malignant biliary lesions have been reported to include tortuous vessels with irregular dilatations, easily hemorrhagic mucosa, irregular papillary lesion growth, and submucosal tumor-like nodular elevations [10,18,19]. However, it is difficult to differentiate tumors from non-tumors when they are accompanied by highly inflamed mucosa, and dilated and tortuous vessels may be observed even in inflammatory mucosa and IgG4-related sclerosing cholangitis [9,20,21]. In the case of IgG4-associated cholangitis in the current study, the biopsy did not suggest malignant findings, and biliary tract cancer could not be ruled out through fluoroscopic contrast and the cholangioscopy findings; therefore, surgery was performed. In recent years, the classification of the differential diagnosis of benign and malignant from the endoscopic findings of cholangioscopy has been reported [22]. Malignant findings can be distinguished on the basis of eight criteria: stricture, lesion, mucosa, papillary projections, ulceration, scarring, pronounced pit, and abdominal mucosal vessels [23]. Further studies are needed to establish the optimal diagnostic criteria, including higher image quality and special light and magnification functions. Moreover, improved operability, larger diameter forceps, and improved durability may lead to improved diagnosis and treatment results in the biliary tract and the development of new treatment methods.

This study has several limitations because of its single-centered, retrospective design. The number of patients was limited, and the number of benign cases was small. Furthermore, we could not eliminate a certain degree of selection bias. Significantly, the indication of cholangioscopy is unclear. Moreover, there is no standardized management of biopsy for biliary strictures. In particular, the number of specimens to be collected depends on each case or the operator’s decision. In the cases without surgery, the method of follow-up for each case is different, suggesting that the diagnosis may be inaccurate. Because of these limitations, the results of our study should be interpreted carefully.

## 5. Conclusions

Cholangioscopy may be effective in the diagnosis of biliary tract stenosis when it is difficult with a conventional biopsy using ERCP. Differentiating some inflammatory lesions of the bile ducts from malignancy is often challenging, and further research and development of materials are needed to improve the quality of diagnosis using cholangioscopy.

## Figures and Tables

**Table 1:** Comparison of the background between cases with conventional biopsy by ERCP and cholangioscopy.

Category	Conventional Biopsy by ERCP (*n* = 172)	Cholangioscopy (*n* = 15)	*p*-Value
Age in years, median [range]	72.2 ± 11.2	69.7 ± 10.4	0.40
Sex			1.00
- Male	111	10	
- Female	61	5	
Location			0.79
- Hilar region (%)	74 (43.0)	7 (46.7)	
- Distal region (%)	98 (57.0)	8 (53.3)	
Lesion			0.22
- Benign (%)	42 (24.4)	6 (40.0)	
- Malignancy (%)	130 (75.6)	9 (60.0)	

**Table 2:** Details of patients who underwent cholangioscopy.

Case	Age	Sex	Type of Cholangioscope	Endoscopic Diagnosis	Biopsy Obtained under Visualization	Surgical Operation	Final Diagnosis	Accuracy
1	70	M	CHF-B260	Cholangiocarcinoma	N	Y	Cholangiocarcinoma	Y
2	72	M	SpyGlassDS	Cholangiocarcinoma	Y	Y	Cholangiocarcinoma	Y
3	83	M	CHF-B260	IgG4SC	Y	Y	IgG4SC	Y
4	72	M	CHF-B260	Compression by IPNB	N	Y	IPNB	Y
5	77	M	CHF-B260	Inflammatory change	N	N	Inflammatory change	Y
6	49	F	SpyGlassDS	Inflammatory change	Y	N	Inflammatory change	Y
7	49	F	SpyGlassDS	Cholangiocarcinoma	Y	N	Cholangiocarcinoma	Y
8	76	M	SpyGlassDS	Cholangiocarcinoma	Y	Y	Cholangiocarcinoma	Y
9	78	M	SpyGlassDS	Cholangiocarcinoma	Y	Y	Cholangiocarcinoma	Y
10	69	F	SpyGlassDS	Cholangiocarcinoma	Y	Y	Cholangiocarcinoma	Y
11	56	M	SpyGlassDS	Cholangiocarcinoma	Y	Y	Cholangiocarcinoma	Y
12	70	F	SpyGlassDS	Cholangiocarcinoma	Y	Y	Cholangiocarcinoma	Y
13	70	M	SpyGlassDS	Cholangiocarcinoma	Y	Y	Cholangiocarcinoma	Y
14	76	F	SpyGlassDS	PSC	Y	N	PSC	Y
15	78	M	SpyGlassDS	IgG4SC	Y	N	IgG4SC	Y

IgG4SC, IgG4-related sclerosing cholangitis; IPNB, intraductal papillary neoplasm of the bile duct; PSC, primary sclerosing cholangitis; Y, yes; N, no.
